# Dataset of transcribed Indonesian stand-up comedy videos with audience laughter annotations from Kompas TV’s YouTube channel

**DOI:** 10.1016/j.dib.2025.112079

**Published:** 2025-09-19

**Authors:** Supriyono, Aji Prasetya Wibawa, Suyono, Fachrul Kurniawan, Andri Pranolo, Nahdi Saubari, Kunfeng Wang

**Affiliations:** a Department of Electrical Engineering and Informatics, Faculty of Engineering, Universitas Negeri Malang, Jl. Semarang no 5, Malang 65145, Indonesia; b Department of Indonesian Literature, Faculty of Letters, Universitas Negeri Malang, Indonesia; c Informatics Engineering, Faculty of Science and Technology, Universitas Islam Negeri Maulana Malik Ibrahim Malang, Indonesia; d Informatics Department, Universitas Ahmad Dahlan, Yogyakarta, Indonesia; e Beijing University of Chemical Technology, No 15 Beisanhuan East Road Chaoyang District, Beijing, China

**Keywords:** Indonesian stand-up comedy, Laughter annotation dataset, YouTube transcription corpus, Humor detection NLP, Spoken language resources

## Abstract

This dataset presents a large-scale compilation of Indonesian stand-up comedy video transcripts collected from Kompas TV’s official YouTube channel. A total of 3934 videos were processed, capturing over 2.8 million words, 6124 sentences, and 17,394 annotated audience laughter events. Each entry includes the video title, URL, the number of laughter instances, the original transcript, and a cleaned version suitable for downstream natural language processing (NLP) tasks. Data collection employed Python-based web scraping, followed by pre-processing routines such as timestamp and tag removal, whitespace normalization, and character cleaning. The dataset supports research in humor detection, speech emotion recognition, and cultural studies of performative discourse in Indonesian. It is particularly valuable for low-resource language NLP development and training models on informal spoken content. Researchers may utilize the dataset for sentiment analysis, summarization, laughter prediction, and sociolinguistic exploration. This openly accessible resource is hosted on Mendeley Data and adheres to ethical standards, with no personal identifiers and full compliance with platform redistribution policies. The dataset fills a notable gap in Indonesian language corpora, particularly in the entertainment and humor domain, providing a foundation for both academic and applied research in computational linguistics and human-cantered AI.

Specifications Table

**Table:** 

Subject	Computer Sciences
Specific subject area	*Natural Language Processing, Humor Analysis, Indonesian YouTube Corpus.*
Type of data	*Text*
Data collection	Web scraping via Python (Beautiful Soup, Requests), transcript pre-processing with regex, collected from Kompas TV YouTube channel.
Data source location	Kompas TV Official YouTube Channel, Jakarta, Indonesia.
Data accessibility	Repository name: Mendeley Data Data identification number: (10.17632/85xgdr7cc7.1) Direct URL to data: https://data.mendeley.com/datasets/85xgdr7cc7/2 Repository name: Mendeley Data Instructions for accessing these data: Click on the direct URL, then use the ‘Download all files’ option in the upper-right corner to obtain the full Excel dataset. No login or special permission is required. The dataset is openly licensed for academic and non-commercial use.
Related research article	[1] Supriyono, A. P. Wibawa, Suyono, and F. Kurniawan, “Analyzing Audience Sentiments in Digital Comedy: A Study of YouTube Comments Using LSTM Models,” J. Appl. Data Sci., vol. 5, *no 4, pp. 1877–1889, 2024,* doi: 10.47738/jads.v5i4.393.

## 1 Value of the Data

• This dataset enriches a rare and structured resource for studying humor and laughter in the low-resource language of Indonesian, through its English translation, via over 3900 transcribed stand-up comedy performances.
• Worldwide researchers in natural language processing can reuse this dataset to develop and evaluate models for sentiment analysis, emotion detection, and spoken discourse summarization in informal speech settings.
• The laughter annotations embedded within the transcripts make the dataset particularly valuable for training machine learning models in humor recognition and audience reaction prediction.
• Linguists and sociocultural researchers around the world can analyze the linguistic features, dialect variations, and narrative structures of Indonesian comedic discourse.
• The dataset’s clean format and structured metadata enable seamless integration with text processing pipelines and make it easy to filter and analyze content by show, comedian, or audience response.
• Global developers of assistive technologies can use this dataset to create inclusive language models or educational tools that reflect authentic spoken Indonesian and comedic contexts.

## 2 Background

Indonesian, known as Bahasa Indonesia, is the official language of the Republic of Indonesia. Geographically, it is spoken across the vast archipelago of Indonesia. With over 200 million speakers, it serves as a lingua franca for a nation with over 700 distinct local languages. This linguistic diversity influences spoken Indonesian, leading to various accents and dialects, although the standardized language taught in schools and used in media provides a common ground. The language uses the 26 letters of the Latin alphabet, making it accessible for text-based computational analysis.

Grammatically, Indonesian has several notable features relevant to NLP. It is an agglutinative language, often using prefixes and suffixes to form words. Unlike many European languages, it does not have grammatical gender or verb conjugation for tense, person, or number, which simplifies certain NLP tasks. The typical sentence structure is Subject-Verb-Object (SVO), like English, although word order can be flexible in informal or spoken contexts, as is common in the stand-up comedy performances captured in this dataset.

The creation of this dataset was driven by the increasing demand for organized linguistic resources in humour research, especially for low-resource languages like Indonesian. Existing language corpora, as evidenced in stand-up comedy, predominantly focus on formal or written contexts, and seldom capture spontaneous, performative speech [1,2]. Indonesian stand-up performances, particularly those showcased on Kompas TV’s YouTube channel, are a valuable and underexploited data source that encapsulates informal spoken discourse, regional linguistic variety, and immediate crowd reactions [3]. The methodological basis for assembling this dataset is rooted in natural language processing (NLP), whereby annotated transcriptions are essential for developing and accessing tasks, including sentiment analysis, speech emotion recognition, and humour detection [[4], [5], [6]]. This dataset was created with a research study examining deep learning models for summarising comedic content and forecasting audience laughing [7]. The data article enhances the research by publicly offering the comprehensive dataset utilized for experimentation, accompanied by thorough documentation and pre-processing specifics, to facilitate reproducibility and reuse by other scholars and practitioners addressing analogous issues in linguistics, AI, or media analytics.

## 3 Data Description

Indonesian is the national language of Indonesia, spoken by over 200 million people across the archipelago and the global diaspora. It uses the Latin alphabet with 26 letters and coexists with >700 local languages, including major dialectal influences such as Javanese, Sundanese, Batak, and Bugis. Indonesian grammar is characterized by a subject–verb–object word order, an agglutinative system with affixes that modify word meaning, and relatively simple morphological rules. The language is also highly adaptive, integrating vocabulary from regional and global sources.

The dataset is publicly accessible as a single Excel file in the Mendeley Data repository: https://data.mendeley.com/datasets/85xgdr7cc7/1. The dataset comprises 3934 rows, each corresponding to an individual Indonesian stand-up comedy video from Kompas TV’s YouTube channel, accompanied by detailed annotations of crowd laughter and preprocessed transcripts for natural language processing (NLP) applications.

Table 1 shows the file and folder structure of the dataset as it appears in the Mendeley Data repository. The dataset is organized at a single folder level and contains one primary file named 97_Preprocessed_StandUp_Comedy_Dataset.xlsx. This Excel file contains all the annotated entries and metadata generated by the data collection and processing pipeline. It serves as the central data source for researchers, containing transcript data, laughter annotations, and cleaned text fields structured in a tabular format. No additional subfolders or supplementary raw data files are included, ensuring a compact and easily accessible format for direct analysis or integration into natural language processing workflows.

**Table 1:** File and folder structure.

Folder Level	File / Folder Name	Description
Root	97_Preprocessed_StandUp_Comedy_Dataset.xlsx	Main data file containing transcribed and annotated entries

**Fig. 1:**
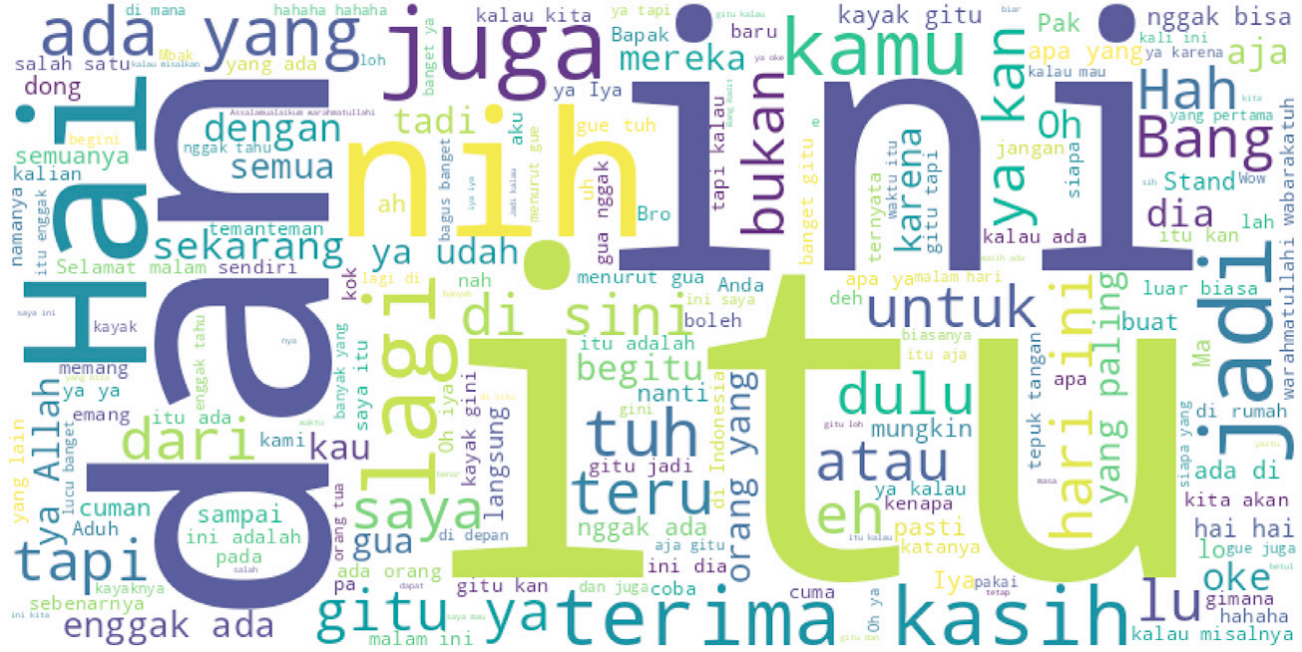
Wordcloud from cleaned transcripts.

The dataset comprises 3934 annotated entries, each corresponding to one stand-up comedy video. For clarity, the dataset is captioned as follows: Dataset of annotated Indonesian stand-up comedy performances from Kompas TV’s YouTube channel, including transcripts, laughter events, metadata, and cleaned text fields for NLP tasks. To support non-Indonesian users, Fig. 1 (Word Cloud) has been supplemented with an English translation of the most frequent words. Table 2 lists the 20 most common tokens and their English equivalents. Example: anak(child); ketawa(laugh); hidup(life); teman(friend).

**Table 2:** English translations of the 20 most frequent tokens.

Indonesian Word	English Translation	Notes on Usage
ini	This	Very frequent demonstrative pronoun in spoken Indonesian.
itu	that, it	Common demonstrative pronoun, used in many contexts.
lagi	again, still	Indicates repetition or continuity.
yang	that, which, who	Relative pronoun, extremely common.
di	at, in, on	Preposition marking location.
juga	also, too	Used for emphasis or addition.
kamu	you	Informal second-person pronoun.
ada	there is/are, to exist	Expresses existence or possession.
ya	yes / (filler word)	Confirmation or discourse marker.
nggak / tidak	no, not	Informal/formal negation.
jadi	so, become	Conjunction (so/therefore) or verb (to become).
hari	day	Refers to time, calendar, or specific day.
untuk	for, to	Preposition indicating purpose or goal.
kasih (terima kasih)	thanks, gratitude	Polite expression, often in phrase terima kasih.
orang	person, people	Refers to an individual or collective group.
anak	Child	Refers to children or offspring.
ketawa	Laugh	Informal term for laughter.
hidup	life, to live	Noun (life) or verb (to live).
teman	Friend	Refers to a friend, companion, peer.
sekarang	Now	Temporal adverb, indicates present time.

Fig. 1 shows a word cloud generated from the Clean_Transkrip column of the dataset. It displays the most frequently occurring words across all cleaned transcripts extracted from Indonesian stand-up comedy performances. The size of each word in the visualization reflects its relative frequency in the corpus, with larger words appearing more often. This representation provides a concise lexical overview of common themes and expressions employed by comedians in the dataset, highlighting informal, humorous, and conversational language patterns characteristic of live comedic discourse.

**Fig. 2:**
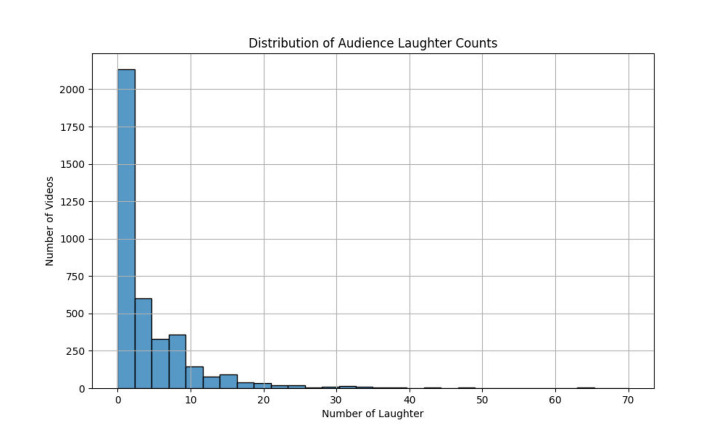
Distribution of audience laughter counts.

Fig. 2 shows the distribution of audience laughter counts (Jumlah Tawa) across all 3934 stand-up comedy videos included in the dataset. The histogram illustrates the frequency of different laughter levels, with most videos containing between 5 and 25 laughter events. A smaller number of videos exhibited very high or very low laughter frequencies, indicating variability in comedic impact or audience engagement. This distribution provides insights into the density and intensity of laughter responses triggered by different performances, offering a valuable reference point for researchers analyzing humor dynamics or training predictive models on audience reactions.

## 4 Experimental Design, Materials and Methods

To aid reproducibility, Figure X illustrates the data collection and preprocessing pipeline. The workflow begins with video scraping and subtitle extraction, followed by transcript cleaning, laughter annotation, and final dataset formatting in Excel. Caption: Workflow of dataset construction showing each stage from YouTube scraping to final dataset release. Additionally, screenshots of sample raw transcripts and cleaned transcripts have been included to demonstrate the preprocessing effect. Handling non-verbal content: Gestural or visual humor was not included in the dataset. Since YouTube transcripts capture only spoken text, gestures and physical expressions remain outside the scope of this dataset. This limitation is explicitly noted to guide researchers interested in multimodal humor analysis.

**Fig. 3:**
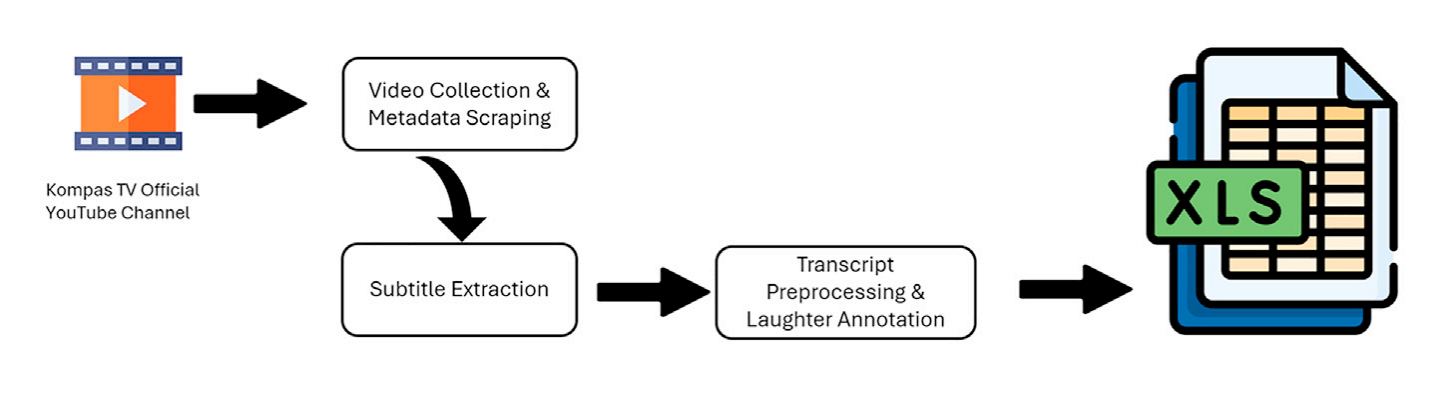
The pipeline from YouTube video scraping to the final structured dataset.

Fig. 3 illustrates the complete workflow of dataset creation, beginning from the Kompas TV official YouTube channel as the primary source. The process starts with video collection and metadata scraping, in which 3934 video URLs and titles were gathered using tools such as Pytube and BeautifulSoup. The next stage involves subtitle extraction with the youtube_transcript_api, producing raw transcripts with timestamps. These transcripts then undergo preprocessing and laughter annotation, performed using Regex and Pandas, which included removing timestamps and tags, normalizing whitespace, and counting laughter markers such as “[tawa].” Afterward, the data were structured and formatted with openpyxl, resulting in a comprehensive Excel dataset containing both raw and cleaned transcripts, video metadata, and laughter counts. This visual representation clarifies the logical flow of the data pipeline and provides readers with a transparent overview of how noisy raw material was systematically transformed into a structured and research-ready dataset.

The dataset was compiled through a structured pipeline consisting of video collection, subtitle extraction, transcript preprocessing, laughter annotation, and dataset formatting. All procedures were conducted using Python-based scripts and standard open-source libraries in a local computing environment.

### 4.1 Video collection and subtitle extraction

Data were obtained from the official YouTube channel of Kompas TV. A total of 3934 stand-up comedy videos were identified and analyzed. Video metadata and subtitle transcripts were obtained utilizing the subsequent Python libraries and APIs:

1. Pytube for obtaining video info, encompassing video ID, title, and URL.
2. Utilization of the YouTube_transcript_api for the extraction of auto-generated YouTube subtitles.
3. Utilizing requests and BeautifulSoup4 for the processing of online content and the extraction of metadata.

*Video URLs were acquired via automated trawling of Kompas TV’s playlist archives. The recovered subtitles were stored in a raw text format, maintaining the original timing and marker annotations.*

### 4.2 Transcript preprocessing

*Raw transcripts were preprocessed to remove noise and prepare the data for NLP tasks. The following preprocessing steps were applied:*

1. Removal of timestamps, speaker labels, and non-verbal cues (e.g., “[Music]”).
2. Normalization of whitespace, punctuation, and letter casing.
3. Retention of audience reaction markers such as [Tawa] for laughter identification.

Regular expressions (regex) and Pandas were used extensively to clean and structure the text. Two versions of the transcript were stored: Transkrip (raw) and Clean_Transkrip (processed). Both were retained in the dataset for versatility across research use cases.

### 4.3 Laughter annotation

Laughter events were automatically annotated by detecting markers such as [tawa], [laugh], or expressive cues (e.g., repeated characters or emphatic punctuation). The number of laughter instances per video was tallied and recorded in the Jumlah Tawa column. This annotation step was embedded into the preprocessing pipeline using string-matching heuristics.

### 4.4 Dataset structure and formatting

The final dataset was stored in an Excel .xlsx file using the openpyxl library. It contains 3934 rows and the following columns (Table 3).

**Table 3:** Dataset structure.

Column Name	Description
No	Unique index number for each entry
Video Title	Title of the stand-up comedy video
Video URL	Direct YouTube URL to the original video
Number of Laughs	Count of audience laughter instances annotated from the video
Transcript	Raw transcript including noise, tags, and timestamps
Clean_Transkrip	Cleaned version of the transcript ready for NLP use
Source File	Indicates the original compilation batch or source file

### 4.5 Software and environment

All scripts were developed and executed using:

1. Programming Language: Python 3.9
2. Operating System: Windows 11 (64-bit)
3. Software Libraries: pytube, beautifulsoup4, youtube_transcript_api, pandas, openpyxl, re, matplotlib, seaborn, wordcloud

Processing was done on a workstation equipped with Intel i7 CPU and 32 GB RAM. All code used in data processing is available upon request to ensure reproducibility.

## Limitations

Several limitations were encountered during the collection and curation of this dataset. First, the dataset is limited to stand-up comedy content published on Kompas TV’s official YouTube channel, which may not fully represent the diversity of Indonesian comedic styles, regional dialects, or informal speech variations found in other performance settings. Second, as the transcripts were sourced from YouTube’s auto-generated subtitles, minor errors such as missing words, misrecognized phrases, and punctuation inconsistencies are possible due to limitations in YouTube’s speech recognition system. Third, laughter annotations were based on detectable textual markers (e.g., “[tawa]”) or heuristic cues; therefore, implicit, subtle, or overlapping audience responses might have been underrepresented. Fourth, variations in video quality, background noise, and overlapping speech in group performances may have affected transcription clarity and annotation precision. Lastly, the dataset does not include speaker segmentation or demographic information, which could be relevant for studies focusing on performer identity, audience composition, or sociolinguistic variation. Despite these limitations, the dataset offers a rare, large-scale, and structured resource for research on Indonesian spoken comedy and audience engagement. Fifth, the dataset excludes gestural or visual humour such as body movements or facial expressions, as YouTube’s auto-generated transcripts only represent spoken language. While this narrows the scope to verbal humour, future multimodal studies may integrate this dataset with video-based gesture recognition resources. Furthermore, the dataset is exclusively text-based, derived from audio transcripts. As a result, it does not capture crucial non-verbal comedic elements such as gestures, facial expressions, or physical interactions from the performances, which are significant components of humour in stand-up comedy.

## Ethics Statement

This work involved data collection from publicly available content on YouTube. The authors confirm that:

a) no personal or sensitive information from individual users was collected or included in the dataset;
b) all videos were sourced from Kompas TV’s official channel, a verified media outlet;
c) data collection complied with YouTube’s Terms of Service and redistribution policies; and
d) no interaction or intervention with human subjects was involved.

All transcripts are automatically generated and openly accessible, and no identifiable personal information about performers or audience members is included in the dataset. The dataset has been fully anonymized and is intended for academic and non-commercial research use only.

The authors confirm that they have read and followed the ethical requirements for publication in Data in Brief and that this work does not involve human subjects, animal experiments, or any data collected from social media platforms that would require informed consent.

## CRediT Author Statement

**Supriyono:** Conceptualization, Data curation, Methodology, Writing – original draft. **Aji Prasetya Wibawa:** Supervision, Validation, Project administration, Writing – review & editing. **Suyono:** Formal analysis, Linguistic annotation, Writing – review & editing. **Fachrul Kurniawan:** Software, Data collection, Visualization. **Nahdi Saubari:** Resources, Quality control, Data verification. **Andri Pranolo:** Investigation, Technical support, Code optimization. **Kunfeng Wang:** Methodology, Cross-linguistic validation, Writing – review & editing.

## Data Availability

Mendeley DataIndonesian Stand-Up Comedy Transcription Dataset (Original data).
